# Intensive glucose control in critically ill adults: a protocol for a systematic review and individual patient data meta-analysis

**DOI:** 10.5935/2965-2774.20230162-en

**Published:** 2023

**Authors:** Derick Adigbli, Li Yang, Naomi Hammond, Djillali Annane, Yaseen Arabi, Federico Bilotta, Julien Bohé, Frank Martin Brunkhorst, Alexandre Biasi Cavalcanti, Deborah Cook, Christoph Engel, Deborah Green-LaRoche, Wei He, William Henderson, Cornelia Hoedemaekers, Gaetano Iapichino, Pierre Kalfon, Gisela de La Rosa, Iain MacKenzie, Christian Mélot, Imogen Mitchell, Tuomas Oksanen, Federico Polli, Jean-Charles Preiser, Francisco Garcia Soriano, Ling-Cong Wang, Jiaxiang Yuan, Anthony Delaney, Gian Luca Di Tanna, Simon Finfer

**Affiliations:** 1 Critical Care Division, The George Institute for Global Health - New South Wales, Australia; 2 Critical Care Medicine, University of Paris - Paris, France; 3 Intensive Care Department, Medical Director of Respiratory Services, King Saud Bin Abdulaziz University for Health Sciences - Riyadh, Saudi Arabia; 4 Department of Anesthesiology, Critical Care and Pain Medicine, Policlinico Umberto I, Sapienza University of Rome - Rome, Italy; 5 Service d’Anesthésie-Réanimation-Médecine Intensive, Groupement Hospitalier Sud, Hospices Civils de Lyon, Pierre-Bénite, France; 6 Department of Anaesthesiology and Intensive Care Medicine, Jena University Hospital - Jena, Germany; 7 Research Institute, HCor-Hospital do Coração - São Paulo (SP), Brazil; 8 Critical Care Medicine, St Joseph’s Healthcare Hamilton - Ontario, Canada; 9 Institute for Medical Informatics, Statistics and Epidemiology, Leipzig University, Leipzig, Germany; 10 Clinical Research, Tufts University School of Medicine -Boston, MA, United States; 11 Department of Critical Care Medicine, Beijing Tong Ren Hospital, Capital Medical University - Beijing, China; 12 VA Emergency Operations Centre, UBC Hospital, University of British Columbia - Columbia, Canada; 13 Department of Critical Care, Radboud University Nijmegen Medical Centre -Nijmegen, The Netherlands; 14 Anestesiologia e Rianimazione, Universitá degli Studi di Milano - Milano, Italy; 15 Hôpital Privé de la Casamance - Aubagne, France; 16 Intensive Care Department, Hospital Pablo Tobon Uribe - Medellin, Colombia; 17 InterSystems Corporation - Cambridge, Mass. , United States; 18 Université Libre de Bruxelles - Bruxelles, Belgium; 19 Australian National University - Canberra, Australia; 20 Department of Anesthesiology and Intensive Care, Helsinki University Hospital and University of Helsinki - Helsinki, Finland; 21 IRCCS Foundation, Cà Granda Hospital - Milan, Italy; 22 Department of Critical Care Medicine, Hospital das Clinicas, Universidade de São Paulo - São Paulo, Brazil; 23 Intensive Care Unit, The First Affiliated Hospital of Zhejiang Traditional Chinese Medical University - Zhejiang, China; 24 Department of Laparoscopic Surgery, The First Affiliated Hospital of Zhengzhou University - Zhengzhou, China

**Keywords:** Blood glucose, Glycemic control, Insulin, Intraoperative period, Mortality, Patient discharge, Registries, Critical illness

## Abstract

**Objective:**

The optimal target for blood glucose concentration in critically ill patients is unclear. We will perform a systematic review and meta-analysis with aggregated and individual patient data from randomized controlled trials, comparing intensive glucose control with liberal glucose control in critically ill adults.

**Data sources:**

MEDLINE®, Embase, the Cochrane Central Register of Clinical Trials, and clinical trials registries (World Health Organization, clinical trials.gov). The authors of eligible trials will be invited to provide individual patient data. Published trial-level data from eligible trials that are not at high risk of bias will be included in an aggregated data meta-analysis if individual patient data are not available.

**Methods:**

Inclusion criteria: randomized controlled trials that recruited adult patients, targeting a blood glucose of ≤ 120mg/dL (≤ 6.6mmol/L) compared to a higher blood glucose concentration target using intravenous insulin in both groups. Excluded studies: those with an upper limit blood glucose target in the intervention group of > 120mg/dL (> 6.6mmol/L), or where intensive glucose control was only performed in the intraoperative period, and those where loss to follow-up exceeded 10% by hospital discharge.

**Primary endpoint:**

In-hospital mortality during index hospital admission. Secondary endpoints: mortality and survival at other timepoints, duration of invasive mechanical ventilation, vasoactive agents, and renal replacement therapy. A random effect Bayesian meta-analysis and hierarchical Bayesian models for individual patient data will be used.

**Discussion:**

This systematic review with aggregate and individual patient data will address the clinical question, ‘what is the best blood glucose target for critically ill patients overall?’

**Protocol version 0.4 - 06/26/2023**

**PROSPERO registration:** CRD42021278869

## INTRODUCTION

Stress hyperglycemia refers to hyperglycemia that commonly accompanies acute and critical illness; it results from increased insulin resistance and glucose production as part of the pronounced endocrine and metabolic response to acute illness. While the association of hyperglycemia with increased mortality has been well known for many years, the concept of intensive glucose control (IGC) in critically ill patients was only investigated following a landmark trial conducted in a single academic center in 2001.^(1)^

In that trial of 1,548 critically ill patients in a surgical intensive care unit (ICU), IGC targeting a blood glucose concentration of 80 - 110mg/dL (4.4 - 6.1mmol/L) reduced morbidity and mortality.^(1)^ Van den Berghe et al. conducted a second trial in 1,200 critically ill patients in the medical ICU of the same medical center and reported that IGC reduced morbidity but not mortality.^(2)^ Based predominantly on the findings of these two studies, the practice of IGC was recommended by many professional organizations and its wide adoption into clinical practice was relatively rapid.^(3)^

The widespread interest in Van den Berghe’s results led other investigators to conduct trials of IGC. The multicenter VISEP trial randomly assigned 488 patients with severe sepsis to IGC or conventional care.^(4)^ The study was stopped before completing planned recruitment because participants assigned to IGC had an increased incidence of hypoglycemia but no reduction in mortality. Additional single-center studies by Arabi et al.^(5)^ and De La Rosa et al.,^(6)^ in mixed surgical and medical ICUs also failed to confirm that IGC reduced mortality. The multicenter GLUCONTROL study was also stopped prematurely and reported that IGC increased the rate of severe hypoglycemia but did not reduce mortality.^(7)^

The 2009 NICE-SUGAR study recruited 6,104 patients from 42 intensive care units (ICUs) in Australia, New Zealand, Canada and the USA.^(8)^ It used a web-based treatment algorithm to target normoglycemia (blood glucose 4.5 - 6.0mmol/L) in comparison with that in the control group (blood glucose < 10.0mmol/L). In contrast to all other studies, the NICE-SUGAR trial reported a significant increase in mortality in patients assigned to IGC compared to those assigned to the control.

A systematic review and meta-analysis published concurrently with NICE-SUGAR and incorporating the NICE-SUGAR study data included 26 trials involving 13,567 patients.^(9)^ In that analysis, the relative risk (RR) of death with IGC was 0.93 (95% confidence interval - 95%CI 0.83 - 1.04), although an apparent benefit of IGC was reported in patients treated in surgical ICUs.^(9)^ An updated systematic review and network meta-analysis published in 2017 included 36 trials involving 17,996 patients.^(10)^ That analysis also failed to find a significant mortality benefit of IGC, with an RR of 0.94 (95%CI 0.83 - 1.07).

Subsequently, Kalfon et al. reported a large multicenter randomized controlled trial (RCT) that compared computerized IGC with CGC and did not find a mortality benefit for IGC.^(11)^ More recently, Bohé et al. reported a multicenter RCT comparing IGC based on individual patients’ glycosylated hemoglobin concentration that reported no mortality benefit from individualized IGC.^(12)^ A post hoc analysis of the study by Bohé found increased mortality in nondiabetic patients assigned to ICG.^(12)^

In September 2023, Gunst et al. reported the provisional results of a large RCT conducted in two hospitals in Belgium that found that intensive glucose control in the absence of early parenteral feeding was not associated with either reduced or increased mortality.^(13)^

### Explanations for discrepancies in the evidence

A variety of hypotheses have been advanced to explain the failure of subsequent trials to replicate the benefits reported in the trials conducted by Van den Berghe et al. At a trial level, these include the use of different target ranges in the control groups, different routes and methods of administering insulin, different sampling sites for the measurement of blood glucose (capillary *versus* whole blood), the use of less accurate glucose meters to measure blood glucose, different feeding strategies and different levels of clinical expertise in the use of IGC protocols.^(14-16)^ Differences with respect to the populations studied, including the proportion of trial participants with preexisting diabetes, may also explain some of the variability in results, as may the use of different control group targets for blood glucose affecting the separation in blood glucose concentrations achieved between groups, differences in other treatments administered in the intensive care unit (e.g., use of concentrated glucose infusions and parenteral feeding), and differences in the duration of follow-up.

### Rationale for an individual patient data meta-analysis

The different effect estimates for IGC in different studies raise important questions regarding optimal glucose control in critically ill adults. Large trials and meta-analyses report average treatment effects in very heterogeneous populations of patients. When the average treatment effect suggests no difference in outcomes between the treatments under study, it is possible that important benefits and harms, that are masked by the heterogeneity of the overall population, may exist for some patient groups.^(17)^ Therefore, we will perform a systematic review and meta-analysis of individual patient data (IPD) to assess whether IGC compared to usual care is associated with reduced hospital mortality both overall and in specific patient groups. We also plan to use this dataset to explore the reasons for the differing effect estimates for IGC in published clinical trials.

## METHODS

### Registration

#### Eligibility criteria

We will include randomized clinical trials in which the entire study population or a clearly identified subgroup within the study population meets the following criteria:

- Critically ill adults, defined as adults being treated in an ICU that can provide invasive mechanical ventilation and advanced organ support to an individual patient for an unlimited period of time.
- Patients in the intervention group will be randomized to a blood glucose target of ≤ 120mg/dL (≤ 6.6mmol/L) using intravenous insulin administration.
- Patients in the comparison (control) group will be randomized to a higher blood glucose concentration target using intravenous insulin administration.
- Blood glucose management according to study protocols could be continued for duration of ICU stay or if stopped after fixed time, that period is at least seven days.
- Mortality at index hospital admission discharge is reported, or mortality can be derived from supplied individual patient data.

We will exclude trials with any of the following characteristics:

- Conducted in coronary care or stroke units.
- Using glucose-insulin-potassium infusions.
- Upper limit of blood glucose target in IGC group of > 120mg/dL (> 6.6mmol/L).
- IGC was performed only in the intraoperative period.
- Loss to follow-up exceeded 10% by hospital discharge.

#### Information sources

We will conduct an electronic search of MEDLINE®, Embase, and the Cochrane Central Register of clinical trials. We will search clinical trial registries (World Health Organization - WHO, clinical trials.gov) to ensure that ongoing trials are not missed. We will search the reference lists of the included studies and relevant review articles and contact experts in the field.

#### Search strategy

We will develop a search strategy consistent with the PRESS guideline statement.^(18)^ We will search using a combination of terms to identify critically ill patients, glycemic control and antidiabetic agents and combine these with sensitivity/specificity filters to refine the search to RCT.^(19)^ Details of the full search strategy are shown in appendix 1.

#### Study records

**Selection of studies and data management:** all records identified by the search will be downloaded into COVIDENCE.^(20)^ Two reviewers will independently screen titles and abstracts. Full text manuscripts will be retrieved for any study adjudicated by either reviewer as potentially eligible. Two reviewers will independently review the full text manuscripts to assess final study eligibility according to the eligibility criteria. Disagreements will be resolved by discussion or adjudicated by a third reviewer. The corresponding author for all included studies will be contacted and asked to provide individual patient data.

**Inclusion of IPDs:** we will include IPDs from any eligible trial where in principle, agreement to share the data has been obtained from the trial’s principal investigator by 1 June 2023.

**Data collection process:** trial-level data will be independently extracted from the included studies by two reviewers. Discrepancies will be resolved by discussion or adjudicated by a third reviewer. The trial-level data to be extracted are shown in appendix 2.

**Individual patient data integrity:** Individual patient data from each participating trial will be comprehensively checked for potential data errors such as spurious values, crucial missing data, dates that do not follow chronological order, and inconsistent information between related data points. Trial data for each trial will be analyzed using the data analysis reported in the trial publication to ensure that the results are reproducible. Any discrepancies identified will be resolved with the corresponding author of the relevant trial. Once all queries are resolved, individual patient data will be merged into a master database for analysis.

### Outcomes

The primary outcome is the proportion of patients who die during index hospital admission.

The secondary outcomes are:

1. Survival analysis to 90 days after randomization.
2. Proportion of patients treated with mechanical ventilation.
2a. Time to alive cessation of mechanical ventilation.^1^
3. Proportion of patients treated with inotropic agents or vasopressors
3a. Time to alive cessation of inotropic agents or vasopressors.^1^
4. Proportion of patients newly treated with renal replacement therapy.
4a. Time to alive cessation of new treatment with renal replacement therapy.^2^
5. Incidence of severe hypoglycemia (blood glucose - BG < 2.2mmol/L).

### Risk of bias

The risk of bias in the included trials will be assessed using the Cochrane Risk of Bias 2 tool.^(21)^ The tool analyses 5 separate domains for bias: (1) arising from the randomization process, (2) due to deviations from intended interventions, (3) due to missing outcome data, (4) in measurement of the outcome, and (5) in the selection of the reported result.^(22)^ A trial will be determined to have an overall high risk of bias if it is judged to have a “high risk” of bias in any single domain or “some concerns” in multiple domains. Two reviewers will independently assess the risk of bias for all included trials, with disagreements resolved by discussion or resort to a third reviewer.

### Data synthesis

#### Main analyses

Two main sets of statistical analyses will be run:

1. Pooling the studies for which IPD were obtained. For these analyses, we will include all trials from which IPD have been obtained.
2. Pooling aggregate data (AD) results including all the results of the eligible studies that were included in the systematic review. For aggregate data analyses, we will include only studies not adjudicated as having a high risk of bias.^(22)^

### Individual patient data meta-analyses

Analyses will use hierarchical models that will include study as a random effect [one-stage approach]. For the main (binary) outcome, we will fit a hierarchical log-binomial model with a random effect at the study level to estimate the pooled RR (along with 95%CI). In case of convergence issues, we will attempt to fit hierarchical Poisson or logistic models (therefore presenting results as incidence rate ratios or odds ratios, respectively).

For the time-to-event analyses, we will fit a shared frailty Cox model with frailty at the study level or a classic Cox model with study as a fixed effect covariate with results presented as hazard ratios (and 95%CI).

Base case models will be based on (hierarchical) univariable regressions, which will include only treatment as a fixed-effect covariate. We will also assess multivariable models to adjust for potential confounding factors, which include the following predefined variables: sex, age, baseline blood glucose concentration, ICU admission type, diagnosis of diabetes mellitus, and severity of illness. To take into account the between-hospital variability in multicenter studies, we will also perform a supplementary analysis fitting a 2-level hierarchical model with study > hospital layers.

We will also assess the robustness of the results by using a two-stage approach, which first calculates summary results of the individual studies as specified below (i.e., reverting to an aggregate data dataset) and then pools these results by an appropriate meta-analytic model. For the latter, we will fit a random effects model based on a Sidik-Jonkman-Hartung-Knapp estimate of the between-study standard deviation (τ).

For binary outcomes, we will use RRs with 95%CI calculated by a univariable log binomial (or Poisson or logistic model in case of convergence issues) for each study with IPD. For time-to-event data, we will use hazard ratios (HRs), which will be calculated by a univariable Cox model for each study with IPD. For time-to-live cessation of mechanical ventilation, inotropic agents or vasopressors, and of new treatment with renal replacement therapy, we will assess subhazard ratios (SHRs) by fitting a competing risks model (death as a competing event).

We will assess quantitative heterogeneity by a formal test of homogeneity and evaluate the proportion of total variability due to heterogeneity rather than by sampling error (I^2^). We will assess small-study effects by regression-based Egger’s test and visual inspection of the contour-enhanced funnel plots. Studies with zero-cell event counts for binary outcomes will be included by using the continuity correction method, which replaces zero event counts with the reciprocal of the sample size of the opposite treatment arm.^(23,24)^

### Bayesian meta-analysis of aggregate data

A Bayesian random-effects meta-analysis of aggregate data results will be performed only for the primary outcome according to the following procedure. The results of the studies for which individual patient data have not been obtained will be used to create a meta-analytic prior distribution for the effect size. This historical/objective prior, combined with a vaguely informative prior for the between-study variance, will inform the Bayesian analyses of the aggregate data results (of the studies for which individual patient data have been obtained). The resulting posterior distribution of the mean effect size will provide the probability that intensive glucose control is associated with a better (or worse) outcome than usual care.

### Subgroup analyses

Subgroup analyses will be performed only for the primary outcome assessed in the individual patient datasets available. We will examine the effect of treatment allocation on index hospital mortality in subgroups defined by patient-level characteristics as well as hospital/study-level characteristics and test for heterogeneity in effects between subgroups. Interpretation of the results will be guided by The Instrument for Assessing the Credibility of Effect Modification Analyses (ICEMAN).^(25)^

### Patient-level subgroups

Patient-level **subgroup analyses** will be conducted on clearly defined and *a priori* baseline characteristics known in individual patients. The following 6 baseline characteristics will define patient-level subgroups/covariates:

**1. Operative versus nonoperative patients:** On theoretical grounds, one could speculate that in surgical ICU patients, hyperglycemia is of recent onset, while in medically critical illness, the duration of hyperglycemia may be much longer, leading to organ damage beyond full recovery. We hypothesize that a beneficial effect of IGC will be more apparent in surgical patients. Surgical patients will be defined as those admitted to the ICU directly from the operating room or recovery room after an operation. Admission after endoscopic or radiological procedures will be classified as medical admissions.

**2. Patients with known diabetes *versus* those without:** Preliminary post hoc analyses from the Leuven studies indicated that IGC may lead to increased mortality risk in patients with known diabetes compared to reduced risk in patients without known diabetes. We hypothesize that the beneficial effect of IGC will be more apparent in patients without known diabetes than in those with known diabetes. Where possible, known diabetes will be defined as a patient taking oral anti-diabetic medication, insulin or a diagnosis of type II diabetes treated with diet.

**3. Patients with sepsis versus those without sepsis:** The VISEP study did not show a benefit from IGC in this specific population of critically ill patients. We hypothesize that a beneficial effect of IGC will be less apparent in patients with sepsis at baseline.

Patients will be included in this subgroup analysis if they were classified as having sepsis or not at the time of inclusion in the original study. Data from studies that did not classify patients were excluded.

**4. Patients with acute brain injury versus those without:** The brain is probably the most vulnerable organ to either hyper or hypoglycemia. We hypothesize that a beneficial effect of IGC will be more apparent in patients admitted with acute brain injury. Patients with acute brain injury will be those whose admission to the ICU that resulted in their inclusion in an IGC trial was for treatment of trauma with brain injury, intracranial hemorrhage (including subarachnoid hemorrhage), ischemic stroke, meningitis or encephalitis.

**5. Patients treated with systemic corticosteroids at baseline versus those not treated:** Is the treatment effect of IGC different in patients treated with systemic corticosteroids at baseline versus those not treated? We hypothesize that a beneficial effect of IGC will be more apparent in patients treated with systemic corticosteroids at baseline. Corticosteroid therapy increases glucose intolerance and could theoretically influence the treatment effect of intensive insulin therapy.

**6. Subgroups classified according to the severity of critical illness:** Differences in the survival benefit of IGC have frequently been attributed to the severity of critical illness. We hypothesize that the adverse effect of hyperglycemia and therefore the beneficial effect of IGC will be more apparent in patients who are less severely ill. Acute Physiology and Chronic Health Evaluation (APACHE II) or equivalent scores as recorded in study databases will be examined as continuous data in relation to mortality to maximize the analytical power. For subgroup analysis, the severity score will be dichotomized as below the median severity score of the entire population Y/N.

### Hospitalor study-level subgroups

Analysis of predefined prerandomization factors that are known only on a center or study basis. We will analyze the following six hospital-level subgroups/covariates:

**1. Early parenteral feeding policy** (unit strategy to deliver > 400 kcal/day of intravenous glucose in the first 72 hours) versus a strategy for later use of parenteral nutrition or concentrated intravenous glucose (≤ 400 iv glucose kcal/day in the first 72 hours). We hypothesize that a beneficial effect of IGC will be more apparent in patients cared for in intensive care units with an early parenteral feeding policy.

**2. Type of glucose monitoring device:** Classified as (1) predominantly bedside point-of-care (≥ 80% of samples), (2) predominantly laboratory or blood gas analyzer (≥ 80% of samples or (3) mixed point-of-care, laboratory or blood gas analyzer (all others). We hypothesize that a beneficial effect of IGC will be more apparent in patients whose blood glucose measurements were predominantly laboratory or blood gas analyzer measurements.

**3. Site of blood sampling:** Classified as predominantly (1) arterial or central venous (≥ 80% of samples), (2) predominantly capillary (≥ 80% of samples) or (3) mixed (all others). We hypothesize that a beneficial effect of IGC will be more apparent in patients whose site of blood sampling is predominantly arterial or central venous.

**4. Unit experience with IGC:** Stratify units into tertiles by number of patients within the trial treated with IGC. We hypothesize that a beneficial effect of IGC will be more apparent in patients treated in units with more experience with IGC.

**5. Type of insulin-infusing system:** Classified as syringe pump or volumetric infusion system or mixed. We hypothesize that the beneficial effect of IGC will be more apparent in patients treated in units where insulin is delivered by a syringe pump.

**6. Control group target:** classified as intermediate (treatment of hyperglycemia started at BG of 10.0mmol/L or lower value) or high (treatment of hyperglycemia started at BG of >10.1mmol/L or higher value). We hypothesize that a beneficial effect of IGC will be more apparent when compared with a higher control group target.

### Risk of bias across studies

We will assess the potential for small study effects and publication bias by visual inspection of contour enhanced funnel plots.

### Strength of accumulated evidence

We will use the Grading of Recommendations Assessment, Development and Evaluation (GRADE) approach to assess the overall certainty of the evidence for the primary and each of the secondary outcomes.^(26)^ We will present the results in a standard summary of findings table. The certainty of the evidence and our confidence in the effect estimates will be based upon a consensus evaluation of the study designs, study quality, precision and consistency of the effect estimates, and the directness in relation to relevance of the outcomes. We will rate the overall certainty as high, moderate, low or very low for each outcome.

### Study management and dissemination of results

#### Study management and coordination

The project will be managed centrally by a steering committee comprising each of the collaborating investigators and members of the coordinating center secretariat. The secretariat will be based at The George Institute for Global Health in Sydney, and coordination, data storage and analysis will occur at this location. Data storage and security will be performed according to the Institute’s standard operating procedures. A representative from each study will be invited to join the steering committee and have the opportunity to contribute to the design, interpretation, and publication of the results. The confidentiality of the data submitted will be assured to all investigators, and the results from the meta-analyses will not be published without agreement from each individual study investigator. If any study investigator requests it, their data can be removed either entirely from the database or from individual analyses after written notification to the secretariat.

#### Publication policy and data sharing

Publications will be in the name of the Intensive Glucose Control Trialists’ Collaboration, each manuscript will have a writing committee, and lead authors of the trials that contributed individual patient data will be included in the writing committee. Each trialist will retain the right to have their data removed from any analysis or publication if they are unable to approve a final manuscript.

After publication of the initial manuscript, data sharing will be considered in accordance with The George Institute Policy for Data Sharing (https://www.georgeinstitute.org.au/data-sharing-policy)

## Appendix 1 Sample search strategy

### Ovid MEDLINE(R) <1946 to June Week 2 2022>

1. exp Glycemic Control/ 1544
2. glycaemic control.tw. 8760
3. glycemic control.tw. 23203
4. exp Hypoglycemic Agents/ 275132
5. hypoglycemic agent*.tw. 2994
6. hypoglycaemic agent*.tw. 1003
7. antidiabetic agent*.tw. 2795
8. exp Insulin/ 194488
9. insulin*.tw. 360080
10. insuline.tw. 210
11. insulinic.tw. 62
12. insuliniz*.tw. 143
13. insulinis*.tw. 48
14. exp Glycated Hemoglobin A/ 40245
15. glycated hemoglobin*.tw. 8070
16. glycated haemoglobin*.tw. 3757
17. glycosylated haemoglobin*.tw. 2384
18. glycosylated hemoglobin*.tw. 7277
19. HbA1c.tw. 34183
20. exp Blood Glucose/ 177610
21. blood glucose.tw. 69434
22. blood sugar.tw. 10333
23. BSL.tw. 847
24. BGL.tw. 1182
25. exp Critical Care/ 64331
26. critical care.tw. 28157
27. ICU.tw. 60522
28. intensive care.tw. 148471
29. intensive care unit*.tw. 117995
30. critical* ill*.tw. 52577
31. exp Critical Illness/ 36094
32. intensive therapy.tw. 4876
33. 1 or 2 or 3 32167
34. 4 or 5 or 6 or 7 276504
35. 8 or 9 or 10 or 11 or 12 or 13 396052
36. 14 or 15 or 16 or 17 or 18 or 19 or 20 or 21 or 22 or 23 or 24 245322
37. 33 and 34 and 35 and 36 9054
38. 25 or 26 or 27 or 28 or 29 or 30 or 31 or 32 247549
39. 37 and 38 700
40. randomized controlled trial.pt. 569969
41. randomized.mp. 888410
42. placebo.mp. 216938
43. 40 or 41 or 42 947337
44. 39 and 43 198

## Appendix 2 Data fields: Trial level

- Study:
  o First author
- Year:
  o Year of publication
- Country:
  o Each country involved in study
- Centres
  o Number of centres in study
- Setting: Type of ICU
  o Medical vs. surgical vs. mixed
- Intervention:
  o Glucose target
- Control:
  o Glucose target
- Glucose measurement
  o How often:
- Duration of follow-up
  o Mortality outcome
- Outcomes
  o Mortality at hospital discharge or nearest timepoint

## References

[1] Van den Berghe G, Wouters P, Weekers F, Verwaest C, Bruyninckx F, Schetz M (2001). Intensive insulin therapy in critically ill
patients. N Engl J Med.

[2] Van den Berghe G, Wilmer A, Hermans G, Meersseman W, Wouters PJ, Milants I (2006). Intensive insulin therapy in the medical ICU. N Engl J Med.

[3] Angus DC, Abraham E. (2005). Intensive insulin therapy in critical illness. Am J Respir Crit Care Med.

[4] Brunkhorst FM, Engel C, Bloos F, Meier-Hellmann A, Ragaller M, Weiler N, Moerer O, Gruendling M, Oppert M, Grond S, Olthoff D, Jaschinski U, John S, Rossaint R, Welte T, Schaefer M, Kern P, Kuhnt E, Kiehntopf M, Hartog C, Natanson C, Loeffler M, Reinhart K, German Competence Network Sepsis (SepNet) (2008). Intensive insulin therapy and pentastarch resuscitation in severe
sepsis. N Engl J Med.

[5] Arabi YM, Dabbagh OC, Tamim HM, Al-Shimemeri AA, Memish ZA, Haddad SH (2008). Intensive versus conventional insulin therapy: A randomized
controlled trial in medical and surgical critically ill
patients. Crit Care Med.

[6] De La Rosa GC, Donado JH, Restrepo AH, Quintero AM, González LG, Saldarriaga NE, Bedoya M, Toro JM, Velásquez JB, Valencia JC, Arango CM, Aleman PH, Vasquez EM, Chavarriaga JC, Yepes A, Pulido W, Cadavid CA, Grupo de Investigacion en Cuidado intensivo: GICI-HPTU (2008). Strict glycaemic control in patients hospitalised in a mixed
medical and surgical intensive care unit: a randomised clinical
trial. Crit Care.

[7] Preiser JC, Devos P, Ruiz-Santana S, Mélot C, Annane D, Groeneveld J (2009). A prospective randomized multi-centre controlled trial on tight
glucose control by intensive insulin therapy in adult intensive care units:
the Glucontrol study. Intensive Care Med.

[8] Finfer S, Chittock DR, Su SY, Blair D, Foster D, Dhingra V, NICE-SUGAR Study Investigators (2009). Intensive versus conventional glucose control in critically ill
patients. N Engl J Med.

[9] Griesdale DE, de Souza RJ, van Dam RM, Heyland DK, Cook DJ, Malhotra A (2009). Intensive insulin therapy and mortality among critically ill
patients: a meta-analysis including NICE-SUGAR study data. CMAJ.

[10] Yamada T, Shojima N, Noma H, Yamauchi T, Kadowaki T. (2017). Glycemic control, mortality, and hypoglycemia in critically ill
patients: a systematic review and network meta-analysis of randomized
controlled trials. Intensive Care Med.

[11] Kalfon P, Giraudeau B, Ichai C, Guerrini A, Brechot N, Cinotti R, Dequin PF, Riu-Poulenc B, Montravers P, Annane D, Dupont H, Sorine M, Riou B, CGAO-REA Study Group (2014). Tight computerized versus conventional glucose control in the
ICU: a randomized controlled trial. Intensive Care Med.

[12] Bohé J, Abidi H, Brunot V, Klich A, Klouche K, Sedillot N, Tchenio X, Quenot JP, Roudaut JB, Mottard N, Thiollière F, Dellamonica J, Wallet F, Souweine B, Lautrette A, Preiser JC, Timsit JF, Vacheron CH, Ait Hssain A, Maucort-Boulch D, CONTROLe INdividualisé de la Glycémie (CONTROLING)
Study Group (2021). Individualised versus conventional glucose control in
critically-ill patients: the CONTROLING study-a randomized clinical
trial. Intensive Care Med.

[13] Gunst J, Debaveye Y, Güiza F, Dubois J, De Bruyn A, Dauwe D, De Troy E, Casaer MP, De Vlieger G, Haghedooren R, Jacobs B, Meyfroidt G, Ingels C, Muller J, Vlasselaers D, Desmet L, Mebis L, Wouters PJ, Stessel B, Geebelen L, Vandenbrande J, Brands M, Gruyters I, Geerts E, De Pauw I, Vermassen J, Peperstraete H, Hoste E, De Waele JJ, Herck I, Depuydt P, Wilmer A, Hermans G, Benoit DD, Van den Berghe G, TGC-Fast Collaborators (2023). Tight blood-glucose control without early parenteral nutrition in
the ICU. N Engl J Med.

[14] Krinsley JS, Deane AM, Gunst J. (2021). The goal of personalized glucose control in the critically ill
remains elusive. Intensive Care Med.

[15] Van den Berghe G, Schetz M, Vlasselaers D, Hermans G, Wilmer A, Bouillon R (2009). Clinical review: Intensive insulin therapy in critically ill
patients: NICE-SUGAR or Leuven blood glucose target?. J Clin Endocrinol Metab.

[16] Scurlock C, Raikhelkar J, Mechanick JI. (2010). Critique of normoglycemia in intensive care evaluation: survival
using glucose algorithm regulation (NICE-SUGAR)--a review of recent
literature. Curr Opin Clin Nutr Metab Care.

[17] Khan YA, Fan E, Ferguson ND. (2021). Precision medicine and heterogeneity of treatment effect in
therapies for ARDS. Chest.

[18] McGowan J, Sampson M, Salzwedel DM, Cogo E, Foerster V, Lefebvre C. PRESS (2016). Peer Review of Electronic Search Strategies: 2015 Guideline
Statement. J Clin Epidemiol.

[19] Wilczynski NL, Haynes RB, QI Hedges Team (2010). Optimal search filters for detecting quality improvement studies
in Medline. Qual Saf Health Care.

[20] COVIDENCE: Systematic Review Management System.

[21] Sterne JA, Savović J, Page MJ, Elbers RG, Blencowe NS, Boutron I (2019). RoB 2: a revised tool for assessing risk of bias in randomised
trials. BMJ.

[22] Higgins JP, Savović J, Page MJ, Elbers RG, Sterne JA., Higgins JP, Thomas J, Chandler J, Cumpston M, Li T, Page MJ, Welch VA (2023). Assessing risk of bias in a randomized trial. Cochrane Handbook for Systematic Reviews of Interventions. Version 6.4
(updated August 2023). Cochrane.

[23] Sweeting MJ, Sutton AJ, Lambert PC. (2004). What to add to nothing? Use and avoidance of continuity
corrections in meta-analysis of sparse data. Stat Med.

[24] Qijun Li K, Rice K. (2020). Improved inference for fixed-effects meta-analysis of 2 ×
2 tables. Res Synth Methods.

[25] Schandelmaier S, Briel M, Varadhan R, Schmid CH, Devasenapathy N, Hayward RA (2020). Development of the Instrument to assess the Credibility of Effect
Modification Analyses (ICEMAN) in randomized controlled trials and
meta-analyses. CMAJ.

[26] Guyatt GH, Oxman AD, Vist GE, Kunz R, Falck-Ytter Y, Alonso-Coello P, Schünemann HJ, GRADE Working Group (2008). GRADE: an emerging consensus on rating quality of evidence and
strength of recommendations. BMJ.

